# Enhancing online adaptive radiotherapy with uncertainty based segmentation error and out-of-distribution detection

**DOI:** 10.3389/fonc.2025.1637198

**Published:** 2026-01-14

**Authors:** Marissa van Lente, Josien Pluim, Samuel Fransson, Robin Strand, David Tilly

**Affiliations:** 1 Department of Biomedical Engineering, Eindhoven University of Technology, Eindhoven, Netherlands; 2 Department of Medical Imaging, Radboud University Medical Center, Nijmegen, Netherlands; 3 Department of Medical Physics, Uppsala University Hospital, Uppsala, Sweden; 4 Department of Surgical Sciences, Uppsala University, Uppsala, Sweden; 5 Department of Information Technology, Uppsala University, Uppsala, Sweden; 6 Department of Immunology, Genetics and Pathology, Uppsala University, Uppsala, Sweden

**Keywords:** uncertainty estimation, machine learning, radiotherapy, MR-Linac, prostate cancer, Monte Carlo dropout

## Abstract

**Purpose:**

Anatomical segmentation is one of the biggest sources of uncertainty in the online adaptive radiotherapy workflow. The aim of this study was to investigate the relation between the estimated uncertainty in deep learning (DL)-based segmentation and the correctness of the segmentations. In addition, the ability to capture out-of-distribution (OOD) data with uncertainty estimation was tested.

**Materials and methods:**

The Monte Carlo dropout method was applied to estimate the uncertainty of a DL model for magnetic resonance (MR)-guided radiotherapy prostate cancer images, trained to segment the clinical target volume (CTV), bladder, and rectum. The training/validation set consisted of 151 T2 MR scans from 26 patients, while the test set consisted of 65 scans from 10 patients. Predictive entropy (PE) was used to capture predictive (model and data) uncertainty. The PE distributions for correct and incorrect predictions were used to find a threshold value. Predicted segmentations with PE values above this threshold value were allocated to the “uncertain group,” and those below to the “certain group.” Dice scores were computed for both groups, using manual segmentations as ground truth. Mutual information (MI) was additionally used to capture epistemic (model) uncertainty as a means to separate in-distribution (ID) from OOD data. Balanced steady-state free precession MRI scans of 10 healthy volunteers were used as OOD data.

**Results:**

The segmentation model obtained Dice scores of 85.7% for the CTV, 94.8% for the bladder, and 86.6% for the rectum. The highest PE values were found at the segmentation borders. Higher PE threshold values resulted in better separation between the certain and uncertain groups. This shows the ability to detect incorrect predictions with uncertainty estimation. A 100% separation between ID and OOD data was achieved with MI.

**Conclusion:**

Uncertainty estimation from a DL-based segmentation model was seen to correlate with Dice scores for segmentation of MR-guided radiotherapy prostate cancer images. This implies that uncertainty estimation could be used to label the quality of the segmentations in the online adaptive radiotherapy workflow. Preliminary results showed that uncertainty estimation could be used to distinguish between ID and OOD data.

## 1 Introduction

In radiotherapy (RT), one of the biggest sources of uncertainty comes from segmentation uncertainty, regardless of whether segmentation is done manually or with deep learning (DL) (1). An obvious step to reduce erroneous segmentation is to develop a high-performing DL model with high segmentation accuracy. However, DL segmentation models will inevitably not be perfect due to limited training data and observer variability; thus, it is important to study how estimated uncertainties in the model predictions can be of value. For example, erroneous predictions should have high uncertainty values, such that they can be flagged. Consequently, in a human-in-the-loop workflow, clinicians could more readily identify erroneous predictions and manually adjust the segmentations. Incorporating uncertainty estimation, therefore, has the potential to both enhance confidence that radiotherapy is accurately targeted, i.e., ensuring tumor coverage while sparing organs at risk, and improve efficiency by supporting clinicians in refining DL predictions.

Prostate cancer is the most common cancer indication in Sweden,^1^ and radiotherapy is a cornerstone treatment modality for several prostate cancer groups. The most advanced workflow is called online adaptive radiotherapy and is implemented using MR-Linac in Uppsala University Hospital since 2019. This workflow means that diagnostic quality magnetic resonance (MR) images, utilizing the superior soft tissue contrast compared to imaging at conventional treatment machines, are acquired at each daily session with the patient in treatment position. Consequently, the treatment plans are adapted based on the segmentations of the acquired images of the patient in treatment position immediately prior to irradiation. The complex workflow with the patient in treatment position requires fast and accurate segmentation. Supporting clinicians to perform accurate and efficient refinement of the DL segmentation is of utmost importance to make online adaptive radiotherapy available to more patients.

Uncertainty in machine learning can be divided into epistemic uncertainty (EU) and aleatoric uncertainty (AU). EU is also called model uncertainty, as it is a measurement of the uncertainty in the DL model parameters. The EU can be decreased by increasing the training data size, but also by using more homogeneous training and test data. AU, or data uncertainty, comes from observation and scanner noise. Collecting more data does not decrease this type of uncertainty, but increasing the scanner precision does. The combination of EU and AU is called predictive uncertainty, generally portraying the confidence of a prediction (2, 3).

Several methods exist for uncertainty estimation, including Bayesian neural networks (BNNs) that make use of the predictive distributions in neural networks (3). However, Bayesian methods come with high computational costs, partially due to inference (i.e., integrating over model parameters) (3). Variational inference can also be used to approximate the posterior distribution in complex models, but the computational cost remains high (4). Instead, a less computationally expensive method, called the Monte Carlo dropout (MCD), can be used, as it has been proven to be a good approximation of traditional BNNs (4). MCD is easy to implement in most existing segmentation models, as long as the architecture contains dropout layers. Dropout layers were originally introduced to reduce the risk of overfitting by randomly dropping units during training (5, 6). MCD works by activating dropout both at training and inference time and by performing multiple stochastic forward passes through the model, resulting in the generation of stochastic predictions. The stochastic predictions can be seen as samples from a probabilistic distribution, and with enough samples, an estimate of the distribution is obtained. With this probability distribution, the estimated uncertainty of the output can be quantified with various metrics and visualized in uncertainty maps. The dropout probability determines how many neurons are randomly dropped. In turn, the dropout probability influences how well the BNN posterior is approximated, and thus, it also affects the predictive uncertainty (3).

Previous research on uncertainty estimation for segmentation in the field of radiotherapy reveals that high uncertainties were detected at difficult and visually ambiguous structures (7, 8). However, thorough analysis of the correlation between the correctness of the prediction and the estimated uncertainty is often lacking (9). In addition, uncertainty estimation is often only tested on in-distribution test data. When a segmentation model is wrongfully applied to out-of-distribution (OOD) data, e.g., data that significantly differ from the training data, there is a substantial risk of leading to incorrect predictions. These types of errors should also be captured by uncertainty estimation.

The aim of this research was therefore to investigate the relationship between uncertainty estimation using MCD and the correctness of the predicted segmentations for MR-Linac prostate cancer images. In addition, the ability to capture OOD data with uncertainty estimation is tested.

## 2 Materials and methods

### 2.1 Patient data

All patients in the in-distribution (ID) dataset received radiotherapy treatment at the MR-Linac at Uppsala University Hospital in Uppsala, Sweden. The dataset consists of 216 T2-weighted MRI scans of 36 prostate cancer patients. Technical specifications of the scans can be found in **Supplementary Material S1**. Ethical approval was given by the Swedish Ethical Review Authority (2019-03050) to use the data in retrospective studies, such as this one. Informed consent was obtained from all subjects. Each patient received between two and seven fractions. For all scans, the segmentations of the clinical target volume (CTV), bladder, and rectum were taken from the treatment plans and manually corrected by one medical physicist with 5 years of experience. The CTV was delineated as the entire prostate without seminal vesicles or a seminal vesicle base, and it was the target for radiotherapy treatment. The patient data were split into sets of 124 scans from 22 patients, 27 scans from 4 patients, and 65 scans from 10 patients for training, validation, and testing, respectively. The scans were kept paired for each patient.

The out-of-distribution data consisted of steady-state free precession MRI scans of 10 healthy volunteers with manual segmentation of the prostate, bladder, and rectum. Ethical approval was given by the Swedish Ethical Review Authority (2021-00831) to use the data in retrospective studies. Informed consent was obtained from all subjects. The segmentations were made by the same physicist who corrected the patient data. The exact scan settings can be found in **Supplementary Material S3**. An example of the visual difference between ID and OOD scans can be found in **Supplementary Material S5**, **Supplementary Figure S4**.

### 2.2 Uncertainty estimation with Monte Carlo dropout

The uncertainty estimation with Monte Carlo dropout was realized using dropout at training time and at inference time. Multiple predictions from a probabilistic distribution are obtained by passing the input image *T* times through the segmentation network with MCD. The number of stochastic forward passes was set to *T* = 50, following (10), as this led to stabilization of accuracy. The actual model prediction for one input is computed by averaging over the *T* output predictions. This model prediction is given as a probability for belonging to a specific class. The estimated uncertainty for the averaged prediction can be quantified and visualized, for example, with uncertainty maps, by using two uncertainty metrics: predictive entropy (PE) and mutual information (MI). PE captures a combination of epistemic and aleatoric uncertainty, i.e., predictive uncertainty, while MI captures epistemic uncertainty. PE is the entropy of the predictive distribution, and it is computed with Equation 1:

(1) $$\hat{ℍ}=-\sum_{c}\left(\frac{1}{T}\sum_{t}p_{c,{\hat{w}}_{t}}(y|x)\right)\text{log }\left(\frac{1}{T}\sum_{t}p_{c,{\hat{w}}_{t}}(y|x)\right),$$

where *c* ranges over all classes; *T* is the number of stochastic forward passes (Monte Carlo samples); $p_{c,{\hat{w}}_{t}}(y|x)$ is the softmax probability of output *y* being in class *c*, given an input x; and ${\hat{w}}_{t}$ are the model parameters on the *t*th Monte Carlo sample (3). Mutual information is computed with Equation 2:

(2) $$\hat{I}=\hat{ℍ}+\frac{1}{T}\sum_{c,t}p_{c,{\hat{w}}_{t}}(y|x)\text{log }(p_{c,{\hat{w}}_{t}}(y|x)).$$

The MI between the predictive distribution and the posterior over network weights can be expressed as the predictive entropy minus expected entropy (3). The expected entropy is the mean of the entropy of the predictions given the parameters over the posterior distribution (11). For both PE and MI, it holds that a low value equals low uncertainty and a high value equals high uncertainty.

### 2.3 Segmentation model

The U-Net has been shown to perform very well in the field of medical image segmentation (12). In this research, a 2D U-Net architecture with five levels was used for the segmentation of the CTV, bladder, and rectum. Two convolutional layers were applied in every stage. The first layer contained 30 channels. With max pooling, the number of channels was doubled in the consecutive encoding layers to 480 channels in the bottleneck layer. Spatial concrete dropout was employed following each convolution in both the encoder and decoder, resulting in a mean dropout probability of 0.09. This type of dropout was used to find the optimal probabilities with regard to both uncertainty estimation and model accuracy for each layer (13). As an activation function, leaky ReLU was used with a slope of 1e−2. The final layer of the network provided a softmax output, giving a pixel’s probability of belonging to each of the three classes or to the background, which could be considered the fourth class. The model was trained for 20 epochs using a batch size of 8. The Adam optimizer (14) was employed with a learning rate of 1e−4. To improve generalization, data augmentation was applied in the form of left−right flipping with a probability of 50%. The cross-entropy loss function was used during training. More details about the hyperparameter settings can be found in **Supplementary Material S2**. The final predicted segmentation masks were obtained by averaging over all 50 Monte Carlo samples per input image and binarizing the averaged masks. Binarization was done by mapping the probability values to one for the class that had the highest probability and zero for the remaining three classes for each pixel. The segmentation model performance is quantified using the Dice similarity coefficient (DSC), the 95th percentile Hausdorff distance (HD95), and the mean surface distance (MSD).

### 2.4 Uncertainty estimation error detection

To investigate the relation between the estimated uncertainty and the correctness of the predicted segmentations, the distributions of the PE per type of classification [i.e., true positive (TP), true negative (TN), false positive (FP), and false negative (FN)] were used for each class. The per-class PE values were computed by omitting the class summation in Equation 1. For example, pixels were classified as TP for the CTV class if they were segmented as CTV in the ground truth and predicted as CTV by the model. TN meant that pixels were correctly predicted as background. FP for the CTV class meant that pixels were falsely predicted as CTV when they belonged to the bladder, rectum, or background class in the ground truth. Lastly, FN meant that pixels were incorrectly predicted as background. With these distributions, threshold values for the PE could be selected to distinguish as good as possible between correct and incorrect predictions. Local (i.e., pixel-based) predictions of the segmentations with PE values above this threshold value were assigned to the “uncertain group” and those below to the “certain group,” similar to the research of Alves et al. (15). Following this separation, the performances within the uncertain group and the certain group were quantified using the DSC by comparing the predictions with the ground truth segmentations. Here, the DSC was computed by making use of the TP, FP, and FN counts per class. If the correctness and the uncertainty of the prediction are indeed linked, the performance within the certain group should be msuch higher than the performance within the uncertain group. As a result, the predictions of the uncertain group could be flagged to focus the attention of the clinician on those pixels that are most likely classified incorrectly.

### 2.5 Out-of-distribution detection

In addition to testing how well segmentation errors can be detected with MCD, the ability to detect OOD data was tested. As described above, the OOD data had a different contrast than the ID data. This choice for OOD data is realistic, since steady-state free precession MRI scans are also used in radiotherapy. Hypothetically, the developed segmentation model could be wrongly applied to these types of scans in practice. With OOD data, the epistemic uncertainty of a segmentation model is expected to increase, which is reflected by a slight increase in PE and a relatively large increase in MI for OOD compared to ID data (16). Thus, to test if these OOD scans can be detected, the trained model was used to predict the segmentations and make global estimates of the uncertainties. The mean MI value (for all classes combined) of each 3D scan was computed for both the OOD and ID data and compared between the groups to explore if they could be distinguished with uncertainty estimation on a global scale.

## 3 Results

### 3.1 Segmentation model performance

The performance of the segmentation model on the in-distribution test set is summarized in **Table 1**. For the CTV, bladder, and rectum, the DSC scores were 85.7%, 94.8%, and 86.6%, respectively. The performance on the out-of-distribution test set can be found in **Table 2**. For the CTV (i.e., healthy prostate), bladder, and rectum, the DSC scores were 64.6%, 78.3%, and 63.7%, respectively.

**Table 1:** Segmentation performance on in-distribution test data.

Metrics	CTV	Bladder	Rectum
DSC (%)	85.65 ± 3.90	94.81 ± 2.46	86.60 ± 4.32
HD95 (mm)	4.43 ± 1.56	3.24 ± 3.96	6.64 ± 5.66
MSD (mm)	1.55 ± 0.50	0.99 ± 0.64	1.39 ± 0.82

CTV, clinical target volume; DSC, Dice similarity coefficient; HD95, 95th percentile Hausdorff distance; MSD, mean surface distance.

**Table 2:** Segmentation performance on out-of-distribution test data.

Metrics	CTV	Bladder	Rectum
DSC (%)	64.55 ± 16.45	78.33 ± 14.37	63.69 ± 13.49
HD95 (mm)	13.34 ± 17.79	4.84 ± 3.11	7.37 ± 3.50
MSD (mm)	2.18 ± 0.93	1.40 ± 0.79	1.97 ± 0.84

CTV, clinical target volume; DSC, Dice similarity coefficient; HD95, 95th percentile Hausdorff distance; MSD, mean surface distance.

### 3.2 Uncertainty threshold for error detection

**Figure 1:**
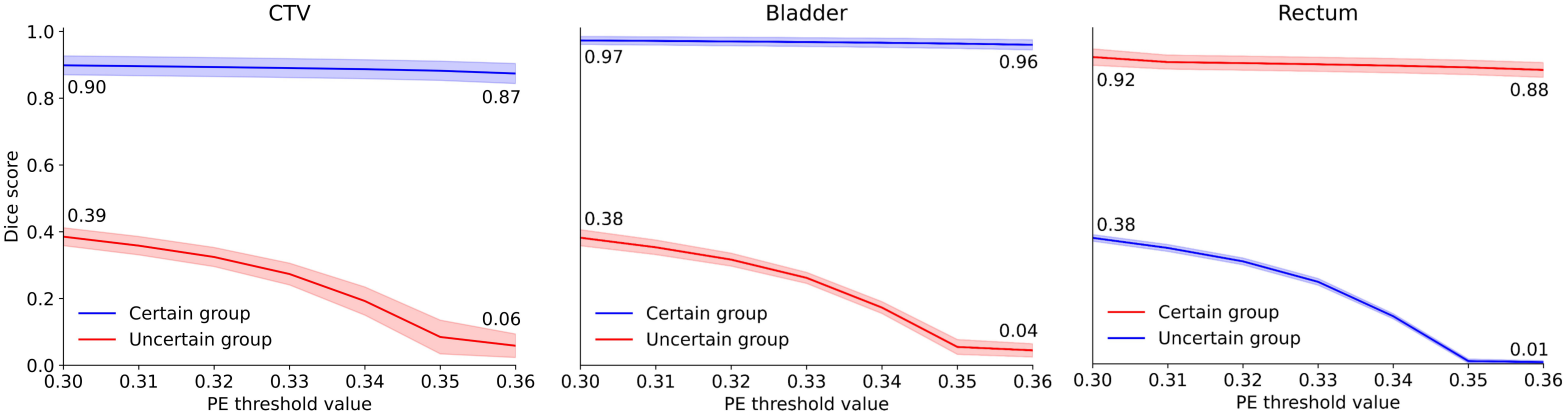
Dice scores for the certain (blue) and uncertain (red) groups with varying thresholds, per class. Shaded areas represent one standard deviation. CTV, clinical target volume; PE, predictive entropy.

The uncertainty of the predictions for the in-distribution data was locally quantified using PE. The pixel-wise PE values were calculated per class. For every pixel, the predicted segmentation was classified as TP, TN, FP, or FN for every class separately. This classification resulted in distributions of the PE values for TP, TN, FP, and FN for each class. The distributions can be found in **Supplementary Material S4**. For all three classes, at least 70% of wrong predictions (FN and FP) had PE values above 0.10, and often more strongly centered around a value of 0.35, with at least 30% having a value of 0.35. At least 97% of correct predictions (TP and TN) had PE values of 0.05 or below. To create groups of certain and uncertain predictions, PE thresholds of 0.30 to 0.36 with increments of 0.01 were selected based on the distinct distributions per classification type. The DSC scores for the certain and uncertain groups with varying thresholds are shown in **Figure 1**, per class. The shaded areas represent one standard deviation. For all thresholds and for all three classes, the performance of the certain group remains much higher than the uncertain group. By increasing the PE threshold, the performance of the uncertain group quickly drops, while it only slightly drops for the certain group. The difference in performance between the two groups thus increases further with higher PE.

### 3.3 Uncertainty maps

**Figure 2:**
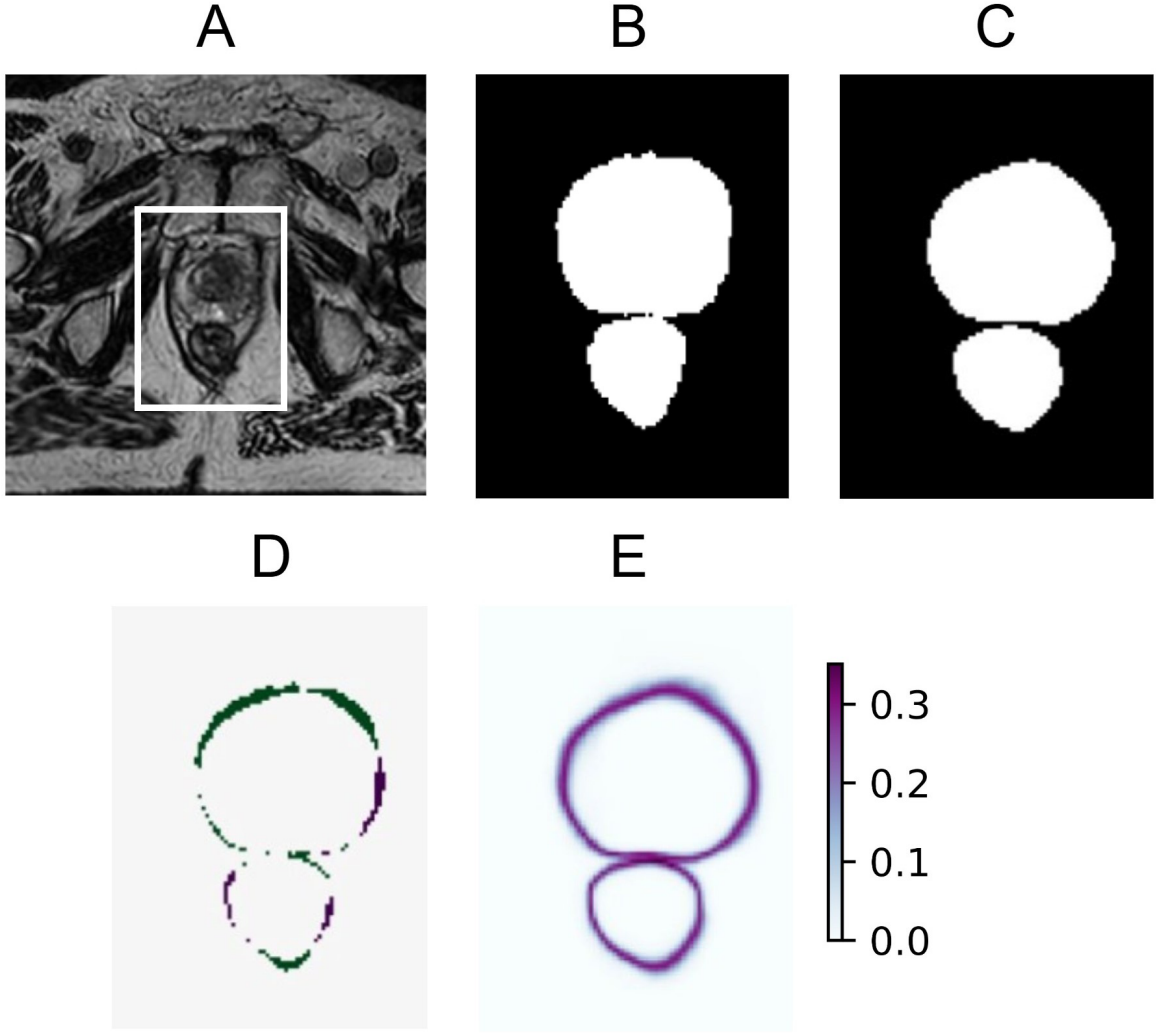
Example of an accurate and certain prediction for the CTV and rectum **(A)**, showing original MRI with a crop box delineating the area magnified in the following panels: ground truth segmentation for the CTV and rectum **(B)**, predicted segmentation for the CTV and rectum **(C)**, prediction error **(D)**, and predictive entropy uncertainty map for all classes **(E)**.

**Figure 3:**
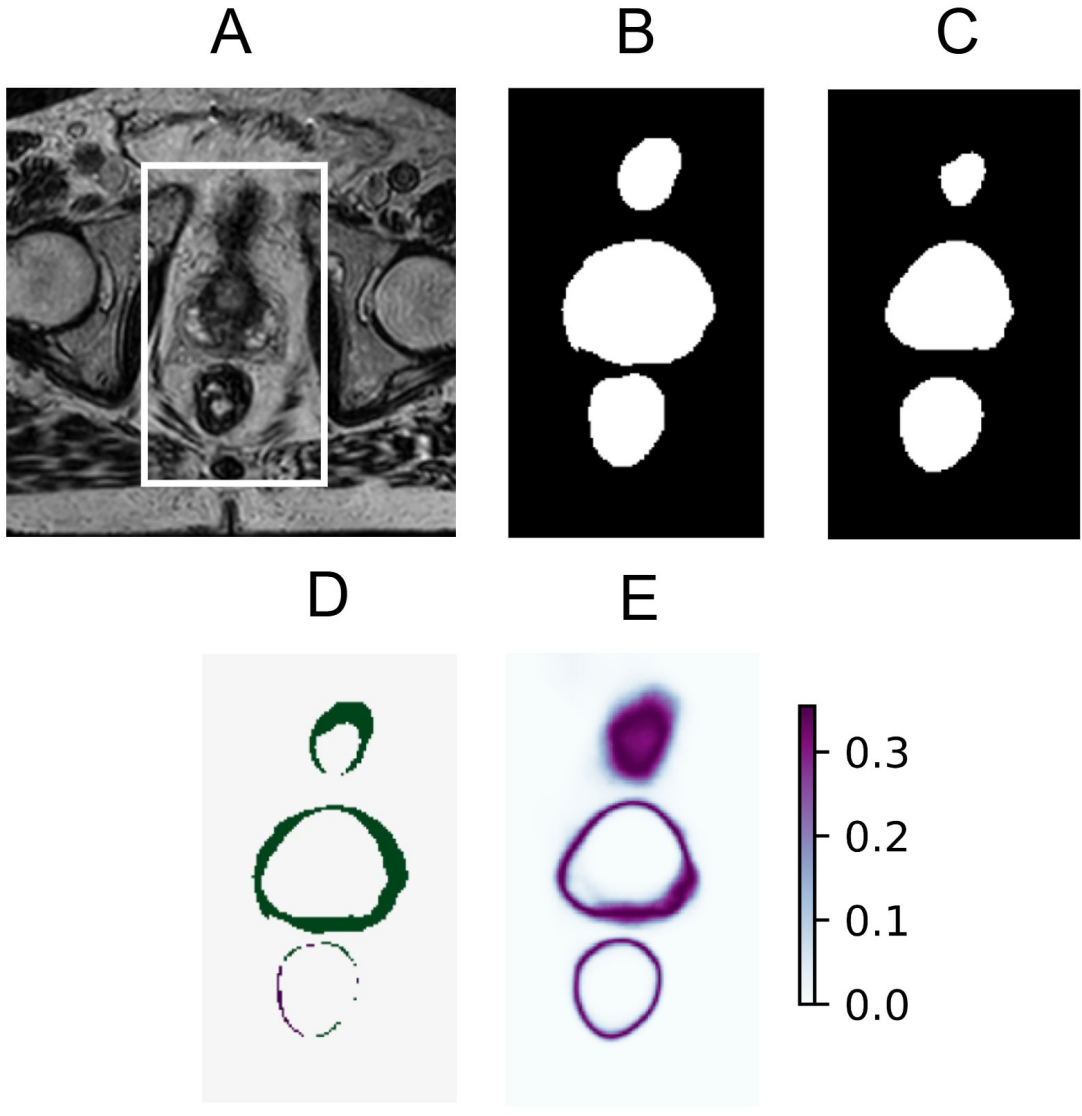
Example of bladder and CTV predictions with erroneous areas and high uncertainty and accurate rectum prediction with low uncertainty **(A)**, showing the original MRI with a crop box delineating the area magnified in the following panels: ground truth segmentation for the bladder, CTV, and rectum **(B)** predicted segmentation for the bladder, CTV, and rectum **(C)** prediction error **(D)** and predictive entropy uncertainty map for all classes **(E)**.

**Figure 4:**
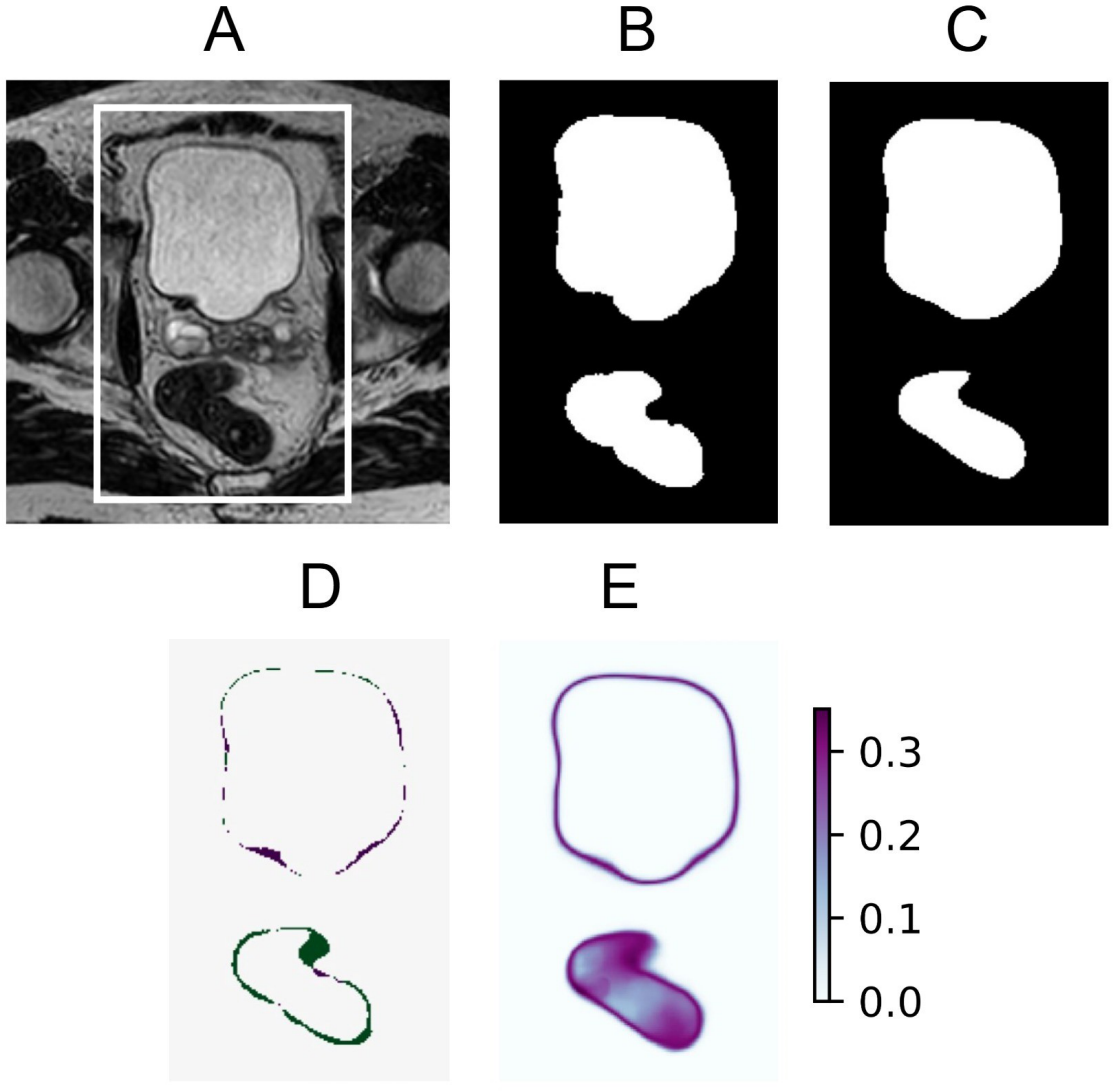
Example of accurate bladder and rectum predictions with large uncertainty for the rectum **(A)**, showing the original MRI with a crop box delineating the area magnified in the following panels: ground truth segmentation for the bladder, CTV, and rectum **(B)** predicted segmentation for the bladder, CTV, and rectum **(C)** prediction error **(D)** and predictive entropy uncertainty map for all classes **(E)**.

The estimated predictive uncertainty could also be visualized using uncertainty maps. Three examples of input images, their ground truth, model predictions, prediction error, and uncertainty map can be seen in **Figures 2**–**4**. The prediction error is the difference between the average prediction after binarization and the manual segmentation. Two examples (**Figures 2**, **4**) contain relatively accurate predictions, while the third example (**Figure 3**) contains more erroneously predicted pixels. In **Figure 2**, the predictions are accurate and low in uncertainty, with the exception of the border pixels. In **Figure 3**, the CTV and bladder predictions are not completely correct. The uncertainty is high in the entire CTV and around the border of the bladder. The rectum is predicted accurately and with low uncertainty. In **Figure 4**, both the bladder and rectum are predicted well, but the uncertainty of the prediction for the rectum is high. In general, the uncertainty maps show higher PE values around the borders of the structures.

**Figure 5:**
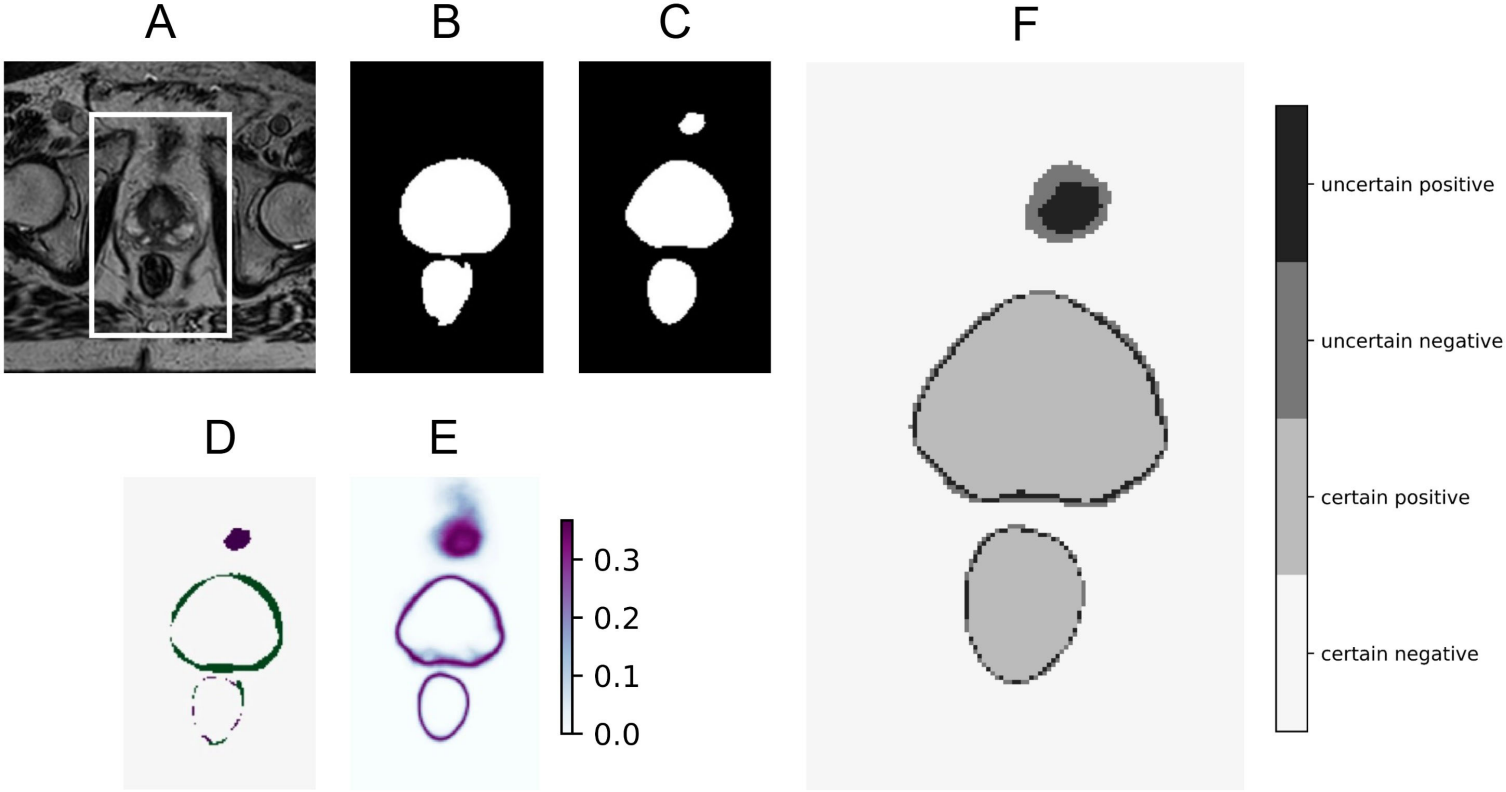
Example of how uncertain pixels may be flagged, showing the original MRI **(A)** with a crop box delineating the area magnified in the following panels: ground truth segmentation for the bladder and rectum **(B)**, predicted segmentation for the CTV, bladder, and rectum **(C)**, prediction error **(D)**, PE uncertainty map for all classes **(E)**, and prediction with pixels flagged as uncertain in the two darkest shades using a predictive entropy threshold of 0.30 **(F)**.

In practice, the uncertainty maps could be used by highlighting all pixels that were categorized as uncertain, after carefully selecting the correct PE threshold for that specific use case. An example of such visualization is shown in **Figure 5F**, together with the original MRI, ground truth segmentation, DL prediction, error in prediction, and full uncertainty map. Here, uncertain pixels are indicated with the two darkest shades. In this example, a PE threshold of 0.30 is used. The darkest shade is used to flag pixels that are predicted as the structure of interest but with high uncertainty, hinting at a potential false positive. The second darkest shade is used to show which pixels are predicted as background with high uncertainty, hinting at a potential false negative. The two lightest shades indicate low uncertainty predictions for the structure of interest (referred to as “certain positive”) and for the background (referred to as “certain negative”).

### 3.4 Out-of-distribution detection

The global mean MI value of every 3D scan was computed for the ID and OOD data. The exact distribution of MI values can be found in **Supplementary Material S5**. The OOD data resulted in significantly higher MI values, portraying higher epistemic uncertainty. A 100% separation between the ID and OOD samples was possible using the mean MI. In addition, the distribution of mean PE values was added in **Supplementary Material S5**, showing that the assumption that PE values also increase for OOD samples holds true. In clinical practice, this would mean that global uncertainty estimation could be applied to distinguish ID from OOD samples. Local uncertainty estimation could then be used to flag potential errors on ID scans.

## 4 Discussion

The current research showed a clear relationship between the estimated uncertainty from DL-based segmentation using Monte Carlo dropout and ground truth segmentation of prostate cancer patient MR images.

It was shown that PE could be used to predict the quality of the DL-based segmentation measured in DSC, on a pixel level. Sorting pixels into uncertain/certain categories based on a PE threshold revealed that the certain group had a significantly higher DSC than the uncertain group.

The performance of the DL segmentation model in this research is comparable to DL models in previous studies on prostate cancer segmentation in MR, with less than 5% difference in DSC scores for all structures (17). Here, the focus was on uncertainty estimation, and therefore, it was not the primary objective to obtain state-of-the-art segmentation results. However, training the segmentation model on 3D rather than 2D input could have increased the performance. By adding cross-slice information, the relationship between the correctness of the DL prediction and the uncertainty could be altered, which should be investigated. This 3D approach could, however, increase the inference time due to the need for multiple forward passes with the MCD method, possibly making it less translatable to clinical use, especially in online adaptive radiotherapy.

The estimated uncertainty offers the possibility to direct the clinician’s attention to areas where special attention is needed, as demonstrated in **Figure 5**. Since segmentation borders are always uncertain, some attention should be directed to them. However, clinicians will always face some ambiguous decision-making about the exact pixels that belong to the borders. It is also important to focus attention on larger areas with high uncertainty, such as shown in **Figure 3**. This has the potential to speed up the editing of the DL-based segmentations, especially important in online adaptive radiotherapy with the patient in treatment position, where segmentation is a time-critical step. This should be explored in a future study to test if segmentation time, including refinement (and possibly quality), improves when uncertain areas are highlighted.

In this study, manual corrections by one medical expert were used as ground truth segmentations. This limits the study as it reduces the validity of the segmentation model’s performance evaluation. However, with multiple experts performing multiple segmentations, random errors are introduced. Capturing these random errors with uncertainty estimation would not be possible. To be able to investigate the relationship between uncertainty estimation and the correctness of the predictions, this type of error was therefore purposely avoided.

The application of the network to out-of-distribution data resulted in significantly higher estimated uncertainties as quantified with mutual information. This suggests that MI could indeed be used as a quality assurance tool to detect when the network is used for segmentation tasks it has not been trained for. However, there are two limitations of the OOD detection analysis. Firstly, the used OOD data differed from the ID data in two ways. The biggest difference is in the contrast settings of the scans, but the use of healthy volunteers for the OOD data also meant that an anatomical difference of the prostate is present. Additionally, patients had larger bladder volumes and a smaller variety in rectum sizes due to a predefined drinking schedule before treatment. Secondly, only one OOD dataset was analyzed. Thus, testing for other data types should be performed before drawing more general conclusions. Datasets with other scan settings, severe imaging artifacts, different patient groups (e.g., rectum patients), or different anatomy (e.g., female pelvis scans) should be additionally tested as OOD data.

Another limitation of this study is the relatively small in-distribution dataset, consisting of 216 scans from 36 patients. By making use of several fraction scans of each patient, this issue is somewhat alleviated, even though the intrafractional variation for a single patient is smaller than the interpatient variability.

## 5 Conclusions

A deep learning-based segmentation model with integrated uncertainty estimation was developed for magnetic resonance images of prostate cancer patients. The results demonstrated that the estimated uncertainty was linked with segmentation correctness, indicating its potential to highlight regions requiring clinical review. Furthermore, the model exhibited significantly higher uncertainty when applied to out-of-distribution data, suggesting its utility in detecting scenarios where the model is used outside its intended use.

## Data Availability

The patient scans used in this research may not be publicly shared and are available upon reasonable request. Requests to access these datasets should be directed to david.tilly@igp.uu.se.

## Footnotes

1
https://www.cancerfonden.se/
