# Supplementary material for: Enhancing online adaptive radiotherapy with uncertainty based segmentation error and out-of-distribution detection
Source: Front Oncol. 2026 Jan 14;15:1637198. doi: 10.3389/fonc.2025.1637198 (PMC12846928; doi:10.3389/fonc.2025.1637198)
Supplement: Supplementary file 1 [file DataSheet1.pdf]

Supplementary Material

1 TECHNICAL SPECIFICATIONS PATIENT DATA

For the fraction scans, which were T2-weighted MR scans, the average repetition time was 1512 ms, the average echo time 263 ms, and the flip angle 90 degrees. The in-plane resolution was equal to 0.86 $\times$ 0.86 mm^2 with a slice thickness of 1 mm. In total 216 scans acquired at the MR-Linac were available. Every 3D scan was converted to 2D transverse data with dimensions of 256 $\times$ 256 pixels for model training and reconstructed back to 3D for data analysis. All 2D images were z-score normalized on the pixel intensity by subtracting the mean value and dividing over the standard deviation.

2 NETWORK HYPER-PARAMETERS

The Monte Carlo concrete dropout U-Net model requires several hyper-parameters to be set. The initial learning rate for the Adam optimizer is set to $1\mathrm{e}{-2}$. The model was trained for 20 epochs until convergence. The used training batch size is 8. For all max pooling layers, a 2 $\times$ 2 pooling window was used. All convolutional layers make use of a 3 $\times$ 3 convolutional window, with an exception of a 1 $\times$ 1 kernel size for the final layer. All upsampling layers make use of transposed convolution. Training of the model was performed on one NVIDIA GeForce RTX 3090 GPU. The deep learning environments Tensorflow 2.4.1 and Keras 2.4.1 were used. Image data augmentation was employed during the training with random left-right flips with 50% probability and random cropping to image size of 192 $\times$ 192.

3 TECHNICAL SPECIFICATIONS OUT-OF-DISTRIBUTION DATA

For the out-of-distribution data scans, a balanced steady-state free precession MRI sequence was used with a repetition time of 5.2 ms, an echo time of 2.6 ms, a flip angle of 60 degrees, and acquisition voxel sizes of 1.5 $\times$ 1.5 $\times$ 2 mm^3.

4 UNCERTAINTY DISTRIBUTIONS PER CLASS

The distributions of the predictive entropy (PE) values for the false and correct predictions are shown per class in Figure S1, Figure S2, and Figure S3.

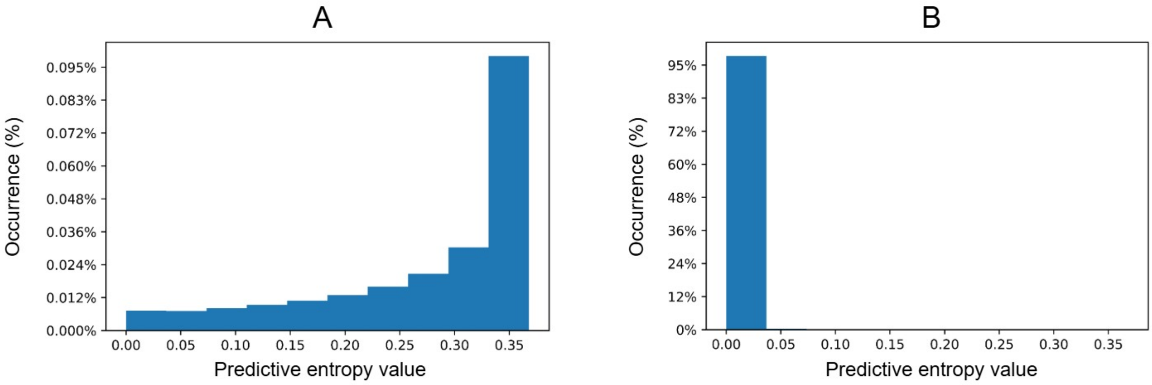

Figure S1. Predictive entropy distribution for bladder pixels, shown for false predictions **(A)** and correct predictions **(B)**.

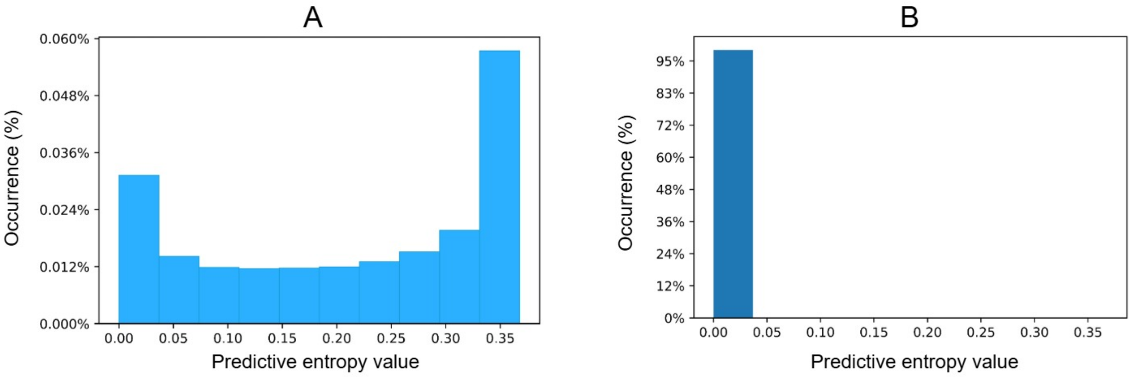

Figure S2. Predictive entropy distribution for CTV pixels, shown for false predictions **(A)** and correct predictions **(B)**.

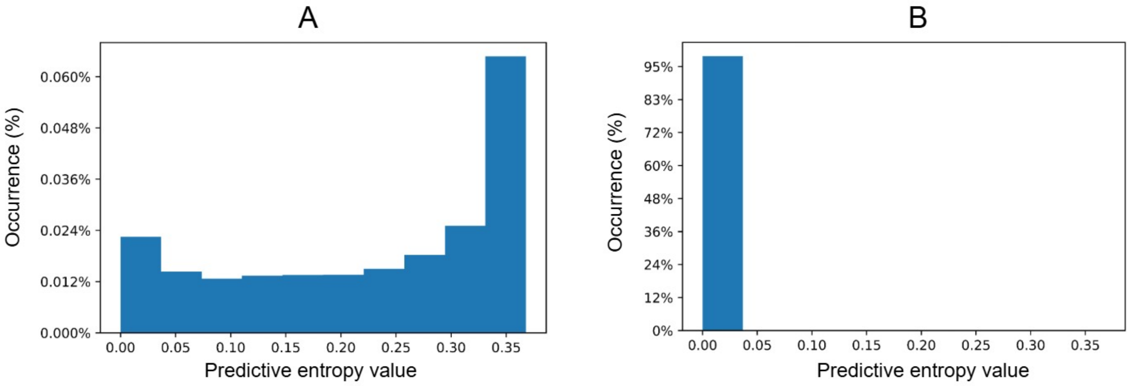

Figure S3. Predictive entropy distribution for rectum pixels, shown for false predictions **(A)** and correct predictions **(B)**.

5 IN-DISTRIBUTION VS. OUT-OF-DISTRIBUTION DATA

Two examples of an in-distribution (ID) and out-of-distribution (OOD) scan are shown to illustrate the differences in Figure S4. The ID data concerns T2-weighted scans of prostate cancer patients. The OOD data concerns balanced steady-state free precession MRI scans of healthy volunteers.

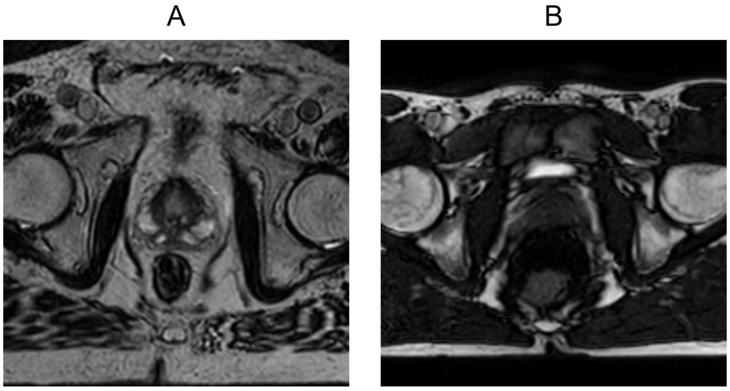

Figure S4. Example slices of in-distribution scan of a patient **(A)** and out-of-distribution scan of a healthy volunteer **(B)**.

Mutual information (MI) has been used as a means of detecting OOD data. In this study, it was shown that the MI achieved 100% separation between ID and OOD samples. For the ID data, the mean MI values ranged between $1.84e{-4}$ and $5.40e{-4}$, with an average value of $3.25e{-4}$. For the OOD data, these values ranged between $5.96e{-4}$ and $1.55e{-3}$, with an average value of $1.09e{-3}$. The entire distribution of the values is shown in Figure S5.

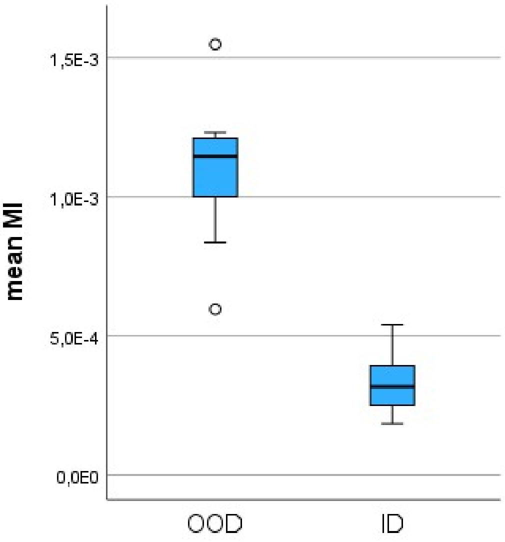

Figure S5. Boxplot of mean mutual information values for out-of-distribution and in-distribution data samples.

Since OOD data should theoretically result in higher epistemic uncertainty, not only the MI values but also the PE values should be higher. To verify if this assumption was true for our data, the mean PE values of the OOD and ID samples were also compared. See Figure S6 for the distribution of these values. Indeed, the OOD samples also have higher PE values. However, completely separating ID and OOD samples with one PE value can not be achieved.

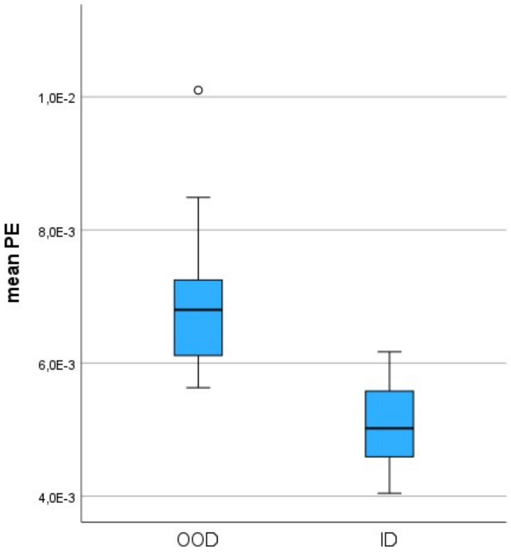

Figure S6. Boxplot of mean predictive entropy values for out-of-distribution and in-distribution data samples.
